# Pharmacological Characterization of Memoquin, a Multi-Target Compound for the Treatment of Alzheimer's Disease

**DOI:** 10.1371/journal.pone.0056870

**Published:** 2013-02-18

**Authors:** Valeria Capurro, Perrine Busquet, Joao Pedro Lopes, Rosalia Bertorelli, Glauco Tarozzo, Maria Laura Bolognesi, Daniele Piomelli, Angelo Reggiani, Andrea Cavalli

**Affiliations:** 1 Drug Discovery and Development, Istituto Italiano di Tecnologia, Genoa, Italy; 2 Department of Pharmacy and Biotechnology, Bologna University, Bologna, Italy; 3 Departments of Anatomy and Neurobiology, Pharmacology and Biological Chemistry, University of California Irvine, Irvine, United States of America; University G. D'Annunzio, Italy

## Abstract

Alzheimer's disease (AD) is characterized by progressive loss of cognitive function, dementia and altered behavior. Over 30 million people worldwide suffer from AD and available therapies are still palliative rather than curative. Recently, Memoquin (MQ), a quinone-bearing polyamine compound, has emerged as a promising anti-AD lead candidate, mainly thanks to its multi-target profile. MQ acts as an acetylcholinesterase and β-secretase-1 inhibitor, and also possesses anti-amyloid and anti-oxidant properties. Despite this potential interest, *in vivo* behavioral studies with MQ have been limited. Here, we report on *in vivo* studies with MQ (acute and sub-chronic treatments; 7–15 mg/kg *per os*) carried out using two different mouse models: i) scopolamine- and ii) beta-amyloid peptide- (Aβ-) induced amnesia. Several aspects related to memory were examined using the T-maze, the Morris water maze, the novel object recognition, and the passive avoidance tasks. At the dose of 15 mg/kg, MQ was able to rescue all tested aspects of cognitive impairment including spatial, episodic, aversive, short and long-term memory in both scopolamine- and Aβ-induced amnesia models. Furthermore, when tested in primary cortical neurons, MQ was able to fully prevent the Aβ-induced neurotoxicity mediated by oxidative stress. The results support the effectiveness of MQ as a cognitive enhancer, and highlight the value of a multi-target strategy to address the complex nature of cognitive dysfunction in AD.

## Introduction

Alzheimer's disease (AD) is a neurological disorder characterized by a progressive loss of cognitive function, dementia and altered behavior. The clinical hallmark of AD is a slow progression from spatial/episodic memory problems to a complete decline of cognitive functions, which leaves patients with late-AD confined to bed and dependent on caregivers, with death occurring a decade after diagnosis [1].

Based on the “cholinergic hypothesis” of AD [2], several classes of acetylcholinesterase (AChE) inhibitors have been identified [3], leading eventually to the discovery of galantamine, donepezil, and rivastigmine. These are the only available drugs for the treatment of AD, apart from memantine, a noncompetitive N-methyl-d-aspartate receptor antagonist. A more recent hypothesis (“amyloid hypothesis”) states that a possible cause of AD is the altered production, aggregation, and deposition of beta-amyloid peptide (Aβ), which results in the formation of Aβ fibrils and plaques [4]. Based on this idea, remarkable efforts have been devoted to the search for disease-modifying drugs. However, to date, no innovative candidates have successfully gone through phase III clinical trials, mainly owing to lack of efficacy or the emergence of toxicity issues [5]. A further and well-recognized neurotoxic pathway in AD is related to the formation of reactive oxygen species (ROS), which can cause cell injury and death. More generally, during aging, oxidative stress is incremented as a consequence of both an accelerated generation of ROS and a gradual decline in cellular antioxidant defense mechanisms [6]. Mitochondria-targeted antioxidants have proven to be successful in counteracting Aβ toxicity in animal models [7] and in improving cognitive function and behavioral deficits in patients with mild to moderate AD [8], [9]. Finally, a further established hypothesis (“tau hypothesis”) states that it is the formation of intracellular neurofibrillary tangles – composed of the hyperphosphorylated form of the tau protein – that plays a major role in AD [10]. Possible interactions between Aβ, oxidative stress, and tangles have also been proposed, making the pathogenesis of this disease extremely complex [11].

In light of the multifactorial nature of AD, it was recently suggested that a polypharmacology-based approach might overcome some of the major limitations of currently available drugs, whose discovery has been based on the one-molecule, one-target paradigm [12]. In particular, a single molecule able to interact with multiple targets thought to be responsible for the disease would present advantages over both single-target drugs and combination therapies [13].

Memoquin (MQ), a quinone-bearing polyamine compound (see Supporting Information, Figure S1, for chemical structure), has recently emerged as a promising anti-AD candidate, mainly due to its multi-target profile [14], [15], [16], [17]. MQ is a nanomolar inhibitor of human AChE, 10 times more potent than donepezil, the most potent anti-AChE drug. MQ has shown a dose-dependent inhibition of spontaneous and AChE-mediated Aβ aggregation [18] and of Aβ_(1–42)_ oligomers-induced neurotoxicity in SH-SY5Y neuroblastoma cells [19]. Furthermore, MQ is able to inhibit in a concentration-dependent manner BACE-1, one of the two enzymes involved in the amyloidogenic cleavage of the amyloid precursor protein. Finally, MQ has antioxidant properties, because it neutralizes the formation of free radicals and ROS in SH-SY5Y cells pretreated with sulforaphane [16].

In the present study, we investigated the effects of MQ *in vivo*, using two different behavioral models, in which amnesia was induced by scopolamine or Aβ. In addition, to shed light on the antioxidant mechanism of MQ, the neuroprotective activity of this compound was investigated in primary cortical neurons, using a model of Aβ-induced neurotoxicity. *In vivo*, MQ was able to rescue several aspects of cognitive impairment. It showed remarkable neuroprotective effects *in vitro*, which may be related to its antioxidant activity.

## Methods

### Animals

Male CD1 and C57BL/6N mice (8 per group, 18–22 g) and female Sprague-Dawley rats (n = 6, for primary cell culture studies) were obtained from Charles River laboratories (Lecco, Italy). After arrival in our facilities, animals were housed in a temperature and humidity controlled room under a 12 h light/dark cycle (lights on at 7 a.m) with water and food *ad libitum*. At least 48 hours before testing, animals were brought to the experimental room and kept in a ventilated storage cabinet (Tecniplast S.p.A, Italy). Acoustic and olfactory stimuli were kept to a minimum. Experimental procedures were performed during the light phase, therefore during the passive phase of the animals.

### Ethics statement

Surgery was performed under chloral hydrate anesthesia, and all efforts were made to minimize suffering. All procedures were performed in compliance with Italian regulations on protection of animals used for experimental and other scientific purposes (D.M. 116192) as well as with European Economic Community regulations (O.J. of E.C. L 358/1 12/18/1986). The protocol was approved by Italian Ministero della Salute (41/2010-B, February 22nd, 2010).

### Drugs and intracerebroventricular surgery

Memoquin (MQ) was suspended in 5% EtOH (Sigma Aldrich, Milano, Italy)/95% water for *per os* (p.o.) administration at various concentrations of 7, 10 and 15 mg/kg (10 ml/kg) and given 40 min prior to behavioral testing. Scopolamine hydrochloride (Sigma Aldrich, Milano, Italy) was dissolved in saline 0.9% and administered intraperitoneally (i.p.) 20 min before testing at 1 mg/kg, 10 ml/kg, unless specified. For β-amyloid i.c.v. injections, mice were anaesthetized i.p. with chloral hydrate (450 mg/kg,10 ml/kg). β-amyloid peptides (1–42 and 42-1) (Bachem, Bubendorf, Switzerland) were dissolved in sterile saline phosphate buffer (PBS), aliquoted at a concentration of 1.8 µg/µl (equivalent to 800 pmol/2 µl) and stored at −20°C. Substances were administered i.c.v. using a microsyringe with a 26s-gauge needle (Hamilton, Bonaduz, Switzerland). In brief, the needle was inserted using a stereotaxic apparatus (Stoelting, Wood Dale, U.S.A.) into the right lateral ventricle, at coordinates 0.20 mm posterior, 1.00 mm lateral to bregma, and 2.50 mm ventral to the skull surface [20]. β-amyloid peptide or sterile PBS (2 µl) were gradually delivered within 2 min. At the end of the experiments, exact injection sites were verified histologically in brain sections using toluidine blue dye to facilitate localizations. Data were analyzed only from those animals that had received injections in the correct target sites. Following i.c.v. injections, animals were randomly divided in 3 different groups receiving daily MQ treatment (15 mg/kg) for 2, 4, 6 days. Behavioral tests were performed starting from day 7 from surgical procedure.

### Behavioral studies

#### Open field

The open field test was performed as previously described [21]. It consisted of a plastic square box 46.5×43.5 cm (Ugo Basile, Comerio, Italy) equipped with an automated activity monitoring system (Any-maze video tracking, Stoelting, U.S.A.). The area of the open field was divided into a 30×28 cm central zone and the surrounding border zone. The experiment was performed under red light. Mice were individually placed into the periphery of the open field and their behavior was tracked. The overall distance travelled by the animals was quantified during an interval of 15 min.

#### Accelerating rotarod

The accelerating rotarod test was performed under red light and as described previously by Morgan and co-authors with minor modifications [22]. Briefly, mice were placed onto the round portion of a motorized circular rod (TSE Systems, Bad Homburg, Germany), which was slowly accelerated starting at 2.5 RPM and reaching 42.5 RPM over 5 min with an increase in speed every 30 seconds. Animals were required to walk at the speed of rod rotation to keep from falling. One trial was performed and the time until falling was recorded for each mouse.

#### Spontaneous alternations

Spontaneous alternation behavior in a T-maze was carried out according to Spowart-Manning and van der Staay [23] with slight modifications. The maze (Ugo Basile, Comerio, Italy) was made of a grey, non-reflective base plate and plastic arms (28×5×10 cm). Training consisted of one single session, which started with 1 forced-choice trial, followed by 14 free-choice trials. In the first trial, the ‘forced-choice trial’, either the left or right goal arm was blocked by a guillotine door made of cardboard. After the animal was released from the start arm, it was allowed to explore the maze, entering the open goal arm, and return to the start position where it would be confined for 5 seconds by lowering the guillotine door. During the following 14 ‘free-choice’ trials and after opening the door, the animal was free to choose between the left and right goal arm. As soon as it entered one goal arm, the other goal arm was closed and once it returned to the start arm, the next free-choice trial started after 5 seconds' restraint in the start arm. A session was terminated and the animal was removed from the maze as soon as 14 free-choice trials were performed or 15 min elapsed. The series of arm entries was recorded visually and the percentage of alternations was calculated as (actual alternations/total possible alternations)×100. The maze was cleaned with a 40% ethanol solution after each session. Animals not finishing the test within 15 min were discarded as considered poorly explorative. The T-maze task was carried out 40 and 20 min after MQ and scopolamine injections, respectively, and on day 7 following surgical i.c.v. procedures.

#### Morris water maze

The Morris water maze (MWM) was performed under red light as described previously [24]. The experimental apparatus (Ugo Basile, Comerio, Italy) consisted of a circular water tank (diameter, 1.2 m; height, 62 cm) filled with water maintained at 22±1°C, placed in a test room containing various prominent visual cues. The target platform (diameter 10 cm, height 31 cm) was submerged 1 cm below the water surface and remained in a fixed position, at the midpoint of one quadrant, throughout the training phase. Training consisted of 2 daily sessions comprising 4 consecutive 60-second trials, each with a 15-second inter-trial interval, during which mice were gently placed in the pool, from various start points, facing the wall and allowed to swim freely to the escape platform. If mice failed to find the platform within the allocated 60 seconds, they were guided to the platform by the experimenter. A trial ended as soon as the animal climbed on the platform and remained on it for at least 2 seconds. After finishing each session, each mouse was allowed to remain on the platform for 20 seconds before being placed in a heated chamber. In order to accelerate the training, an extra trial was added, before the first session, in which mice were placed on the hidden platform for 60 seconds. Animals were trained until they were able to reach the escape platform in less than 20 seconds. The probe session was performed 24 h after the last training session. It consisted of a single probe trial in which the platform was removed from the pool and each mouse was allowed to swim for 60 seconds in the maze. The behavior of the animals was recorded, subsequently elaborated by an automated activity monitoring system (ANY-maze video tracking, Stoelting, U.S.A.) and the percentage of time spent in the platform area was calculated. Treatments with MQ and scopolamine (1.5 mg/kg) were administered 40 and 20 min before the first trial (T1), respectively, before the probe trial only.

#### Novel object recognition

Novel object recognition (NOR) task was conducted according to Bevins and Besheer [25] in an open field box, under red light, on 2 successive days. On the first day, mice were allowed to explore an empty box for adaptation. During this phase, locomotion and basal anxiety states were monitored in order to keep the different experimental groups homogeneous. On the second day, two 5-min trials were performed. Treatments with MQ and scopolamine were before the first trial (T1). In T1, mice were presented with two identical objects called *familiar* objects (F). After 15 min, trial 2 (T2) started and mice were again exposed to two dissimilar objects: the familiar object and a novel object (N). The box floor and the objects were cleaned with a 40% ethanol solution between each animal to eliminate odor contaminations. All trials were video-recorded for manual analysis of object exploration, defined as the time in which a mouse nose touched or was oriented toward the object within 2 cm. The discrimination index was calculated as (N−F)/(N+F).

#### Passive avoidance

To examine emotional (aversive) memory, the step-through passive avoidance task was performed as previously described [26]. Briefly, the apparatus consisted of two compartments connected by a sliding door; one brightly lit and one dark containing a grid floor that could be electrified (Ugo Basile, Comerio, Italy). During the acquisition trial, each mouse was placed in the light compartment and the sliding door was closed. After 20 seconds, the door was opened and the mouse was allowed to enter the dark chamber. The acquisition latency to enter the dark compartment was recorded (in seconds). When the mouse entered the dark compartment (with all four paws), the sliding door was automatically closed and the animal received a mild, unavoidable electric shock (0.8 mA) for 5 seconds. The retention trial was repeated 24 h after the acquisition trial by placing the animal in the light compartment and recording the time taken to enter the dark one (retention latency). Cut-off times of 180 and 300 seconds were used during the acquisition and the retention trial, respectively. Acute MQ and scopolamine treatments were administered before the acquisition trial only and in Aβ-injected mice, the task was performed on days 8–9 following the surgical procedure.

### Statistical Analysis

Data are presented as mean ± standard error of the mean (SEM). Statistical analysis was performed using 1-way ANOVA followed by post-hoc Tukey's test where appropriate. P levels <0.05 were considered statistically significant. Statistical analysis was performed using Graph Pad Prism version 5.00 (Graph Pad Software, San Diego, USA). The ARRIVE guidelines for reporting experiments involving animals were considered in writing the results.

### In vitro studies

#### Reagents

Neurobasal medium, B27 supplement, penicillin/streptomycin and L-glutamine were from Gibco (Paisley, United Kingdom). Cytotoxicity Detection (LDH) and Cell Proliferation (MTT) assay kits were acquired from Roche (Mannheim, Germany). Poly-D-lysine-coated plates were purchased from BD Biosciences (Bedford, MA, USA). L-Sulforaphane and all other reagents were from Sigma (Saint Louis, MO, USA). Aβ_1–42_ peptide was acquired from Bachem (Bubendorf, Switzerland).

#### Cortical neuron isolation

Rat primary neuron cell cultures were obtained using previously described procedures by Agostinho and Oliveira [27] with some modifications. In brief, the neocortices of 17-day embryos from Sprague-Dawley rats were collected and placed in a Ca^2+^ and Mg^2+^-free Krebs buffer. Following trypsinization, the cortices were mechanically dissociated and the Krebs buffer was replaced with Neurobasal (NB) medium supplemented with 2 mM L-glutamine, penicillin (100 U/ml), streptomycin (100 U/ml) and 2% B27. Cell counting was performed using a Nucleocounter NC-100 (Chemometec, Allerod, Denmark) and neurons were plated into 24-well poly-D-lysine-coated plates at a density of 0.25×10^6^ cells per well. The cultures were maintained at 37°C in a humidified atmosphere with 5% CO2/95% air for 5 days in vitro.

#### Cell treatments

Neuronal cultures were treated with sulforaphane for 24 h. Then, the sulforaphane-containing medium was replaced with fresh NB medium and the cells were exposed to Aβ_1–42_ and/or memoquin. Control corresponds to untreated cells. Cell viability was evaluated after 24 h.

#### Neurotoxicity assessment

Neurotoxic damage was evaluated using the MTT and LDH assays according to the kit manufacturers' protocols. Briefly, after cell treatments, MTT (5 mg/ml) was added to the neuronal culture medium for 4 h at 37°C. After this incubation, a solubilizing solution (10% SDS in 0.01 M HCl) was added to the wells and left overnight at 37°C to dissolve the formazan crystals. Absorbance was measured at 570 nm on a Tecan Infinite M200 (Tecan, Männedorf, Switzerland) plate reader.

LDH was measured in the culture medium of treated cells. The sample medium was transferred into a 96-well plate containing the substrate mixture from the kit. LDH activity was determined after 30 min incubation at room temperature in the dark, by measuring absorbance at 490 nm on a Tecan Infinite M200 (Tecan, Männedorf, Switzerland) plate reader. Results were expressed as a percentage of the absorbance in control cells and statistical analysis was performed by One-way ANOVA followed by Dunnett's multiple comparison test using Graph-Pad Prism 5.00 (Graph Pad Software, San Diego, USA).

## Results

### Motor activity

To determine whether MQ influenced locomotor activity in a novel environment as well as motor coordination, we carried out tests in an open field and on accelerating Rotarod. Mice were treated 40 min prior to testing with MQ 7, 10 and 15 mg/kg. Control animals received vehicle. In both tests, no statistically significant differences were observed between MQ- and vehicle-treated animals (see Supporting Information, Figure S2).

### T-maze and Morris water maze

**Figure 1:**
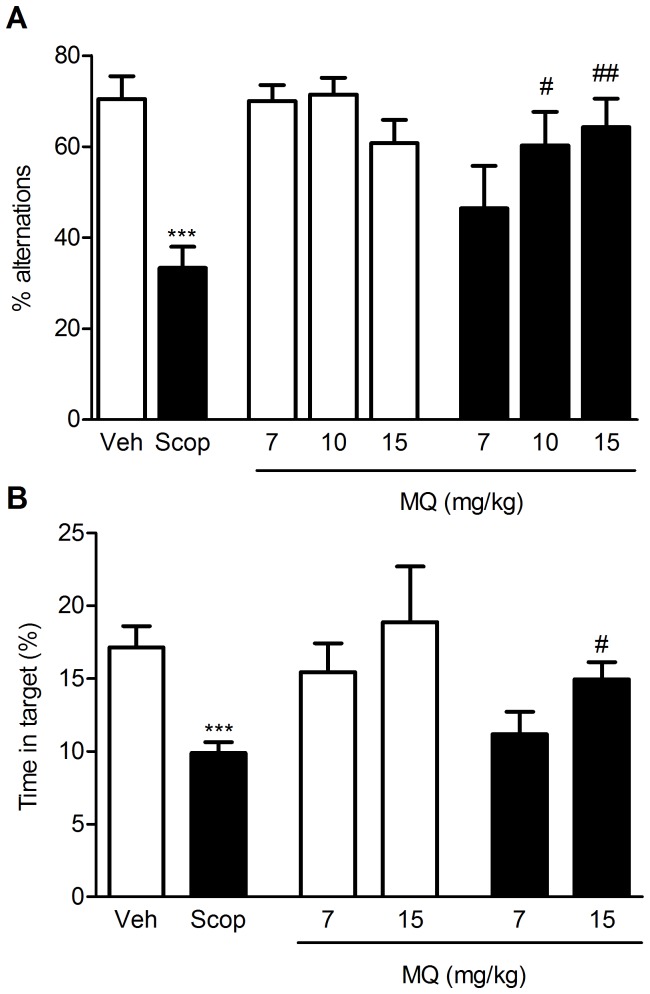
MQ rescues scopolamine-induced spatial memory deficits. Effect of MQ treatment on spontaneous alternation behavior (A) and MWM (B) in a scopolamine-induced amnesia model in mice. In the T-maze, MQ (7–15 mg/kg , p.o.) was administered 20 min before scopolamine (1 mg/kg, i.p.); in the MWM, MQ (7 and 15 mg/kg, p.o.) was administered 20 min before scopolamine (1.5 mg/kg, i.p.). Data are expressed as mean ± S.E.M. Statistical analysis was performed using One-way ANOVA followed by post-hoc Tukey's test where appropriate, *** p<0.001 vs. controls (Veh); ^#^ p<0.05, ^##^ p<0.01 vs. scopolamine-treated animals (Scop).

Spatial memory performance was assessed using the T-maze and the Morris water maze (MWM) tasks. In the T-maze (Fig. 1A), the percentage of alternations positively correlates with the cognitive ability of the animals. Administration of scopolamine (1 mg/kg) produced a significant reduction in the percentage of alternations, compared to vehicle controls (p<0.001). The administration of 10 and 15 mg/kg MQ reversed scopolamine-induced memory deficits (p<0.05 and p<0.01 vs. scopolamine-treated animals at the doses of 10 and 15 mg/kg, respectively). Furthermore, when given in combination with saline (either 10 or 15 mg/kg), MQ did not modify the alternation rate of the mice, compared to vehicle-treated controls. Figure 1B shows the results obtained with the MWM task, expressed as a percentage of time spent in the target area (i.e. where the platform was placed during the training phase) during the probe session. Animals receiving vehicle focused their search in the appropriate area, while scopolamine-treated mice (1.5 mg/kg, i.p.) showed a significant decrease in the time spent in the correct region of the pool (p<0.001 vs. control animals). MQ (15 mg/kg) prevented the negative effect of scopolamine (p<0.05 vs. scopolamine). Conversely, when administered alone, MQ did not alter the animals' performance at either of the doses tested (10 and 15 mg/kg).

### Novel object recognition and passive avoidance

**Figure 2:**
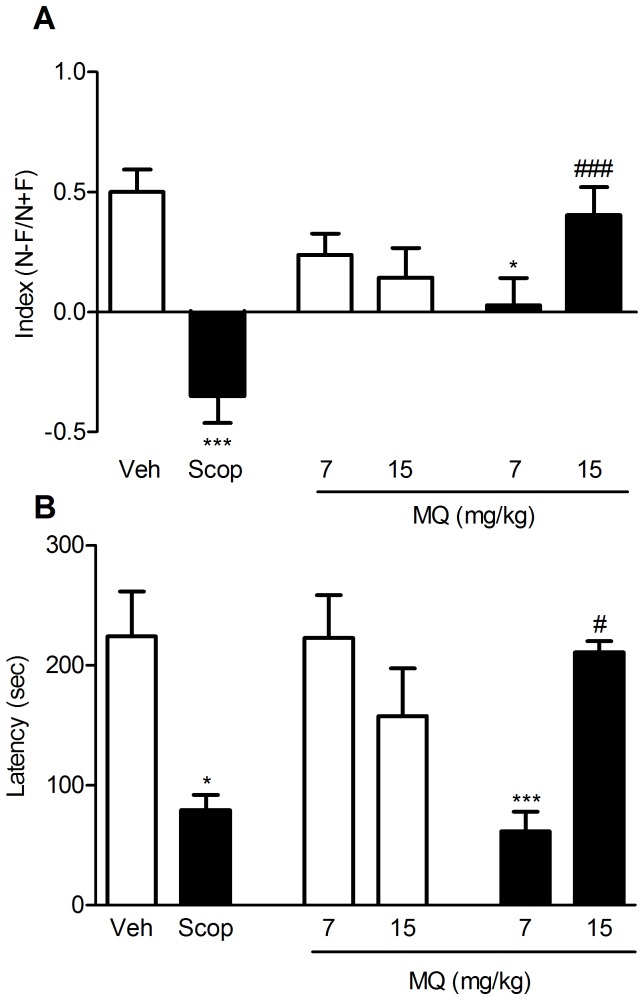
MQ rescues scopolamine-induced episodic memory impairment. Effect of MQ treatment in the novel object recognition (A) and passive avoidance (B) tasks using a scopolamine-induced amnesia model in mice. In both tests, MQ (7 and 15 mg/kg, p.o.) was administered 20 min before scopolamine (1 mg/kg, i.p.) before the first trial only. Data are expressed as mean ± S.E.M. Statistical analysis was performed using One-way ANOVA followed by post-hoc Tukey's test. * p<0.05, *** p<0.001 vs. controls (Veh); ^#^ p<0.05, ^###^ p<0.001 vs. scopolamine-treated animals (Scop).

Episodic memory was assessed using the novel object recognition (NOR) and the passive avoidance tasks (Fig. 2). In NOR, during adaptation to the open field box on day 1, all the animals showed similar locomotor activity and anxiety levels. Furthermore, and independently of the treatment, mice explored equally the familiar objects (F) during T1. Recognition memory of the novel object (N) was investigated and the index of recognition was the behavioral readout. A positive index reflects a good recognition memory between F and N, as shown for the vehicle-treated animals (index = 0.50±0.09, see Fig. 2A). A negative index (−0.35±0.11; p<0.001 vs. controls) was reported for the animals treated with scopolamine (1 mg/kg, i.p.) indicating a poor exploration of N vs. F during T2. The amnesia induced by scopolamine was fully reversed by 15 mg/kg of MQ (p<0.001 vs. scopolamine-treated animals), while MQ did not show any positive effect at a lower dose (7 mg/kg). As a control, when MQ was given in combination with saline, at either tested dose, it did not significantly alter the discrimination index compared to vehicle-treated mice. As for passive avoidance, the effect of MQ on retention latency is reported in Figure 2B. Scopolamine (1 mg/kg, i.p.) caused a strong amnesia in mice, which entered the dark compartment faster (p<0.05) than controls did. This deficit was fully reversed by oral administration of 15 mg/kg MQ (p<0.05 vs. scopolamine-treated animals), while the drug did not show any significant effect at 7 mg/kg. As a control, when MQ was given in combination with saline, at either tested dose, it did not significantly alter the latency compared to vehicle-treated mice.

### β-amyloid injection model

**Figure 3:**
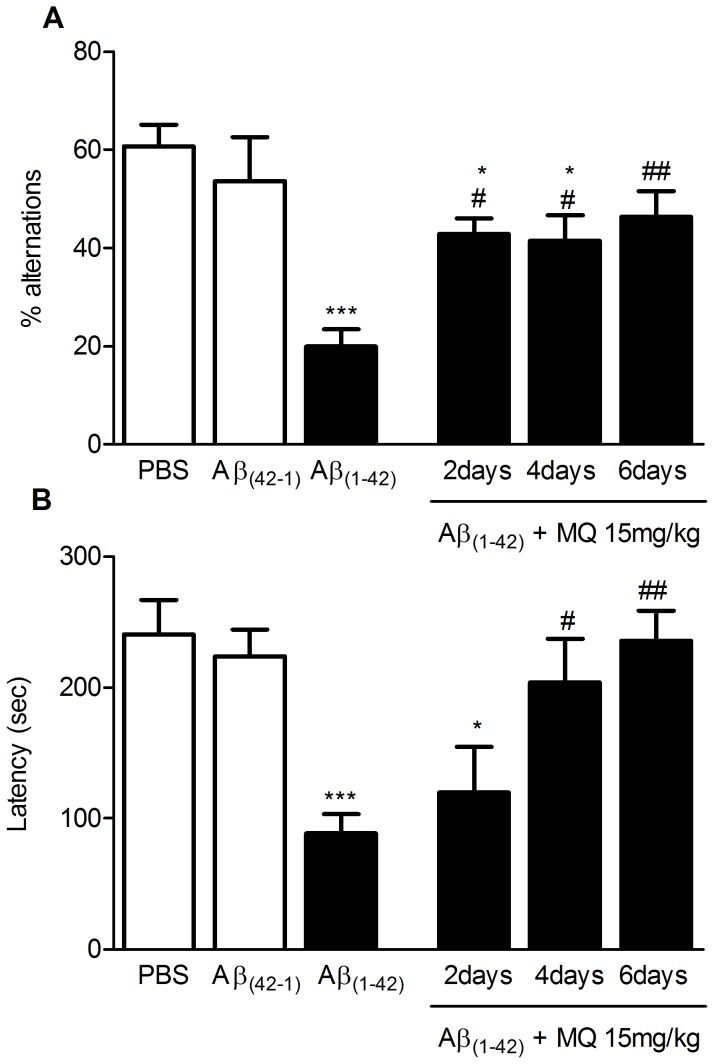
Beneficial effect of MQ on Aβ_1–42_ i.c.v. treated animals. Effect of MQ daily treatments in the spontaneous alternation (A) and passive avoidance task (B) using a Aβ- induced amnesia model in mice. 800 pmol Aβ_1–42_ and Aβ_42-1_ or their vehicle (PBS) were i.c.v. injected 7 and 8 days before T-maze and passive avoidance, respectively. MQ 15 mg/kg p.o. was administered daily for 2, 4, and 6 days before behavioral testing. Data are expressed as mean ± S.E.M. Statistical analysis was performed using One-way ANOVA followed by post-hoc Tukey's test. * p<0.05, *** p<0.001 vs. controls (PBS and Aβ_42-1_); ^#^ p<0.05, ^##^ p<0.001 vs. amyloid-injected animals.

Initial experiments (see Supporting Information, Figure S3) confirmed that intracerebroventricular (i.c.v.) injection of 800 pmol beta-amyloid peptide 1–42 (Aβ_1–42_) in mice produced consistent memory impairment in the T-maze and the passive avoidance tasks. In Figure 3, we report the effect of repeated treatments with MQ (15 mg/kg) on Aβ-induced amnesia using T-maze and passive avoidance. In the T-maze task (Fig. 3A), 800 pmol of Aβ_1–42_, injected i.c.v. caused a significant decrease in the percentage of alternation when compared to controls (p<0.001 vs. controls, i.e. PBS and i.c.v. injection of 800 pmol of beta-amyloid peptide 42-1). While treatment with MQ for 2 and 4 days was able to only slightly tackle the insult by Aβ_1–42_ (p<0.05 vs. Aβ_1–42_, p<0.05 vs. controls), full behavioural rescue was obtained with a 6-day treatment (p<0.01 vs. Aβ_1–42_). Similar results were obtained in the passive avoidance task (Fig. 3B). The retention latency was reduced in Aβ-treated animals (p<0.001 vs. controls). In this case, both 4 and 6-day MQ treatments (15 mg/kg) had significant beneficial effects on amnesia (p<0.05 and p<0.01 vs. Aβ_1–42,_ respectively).

### Aβ_1–42_ toxicity on rat cortical neuronal cultures

**Figure 4:**
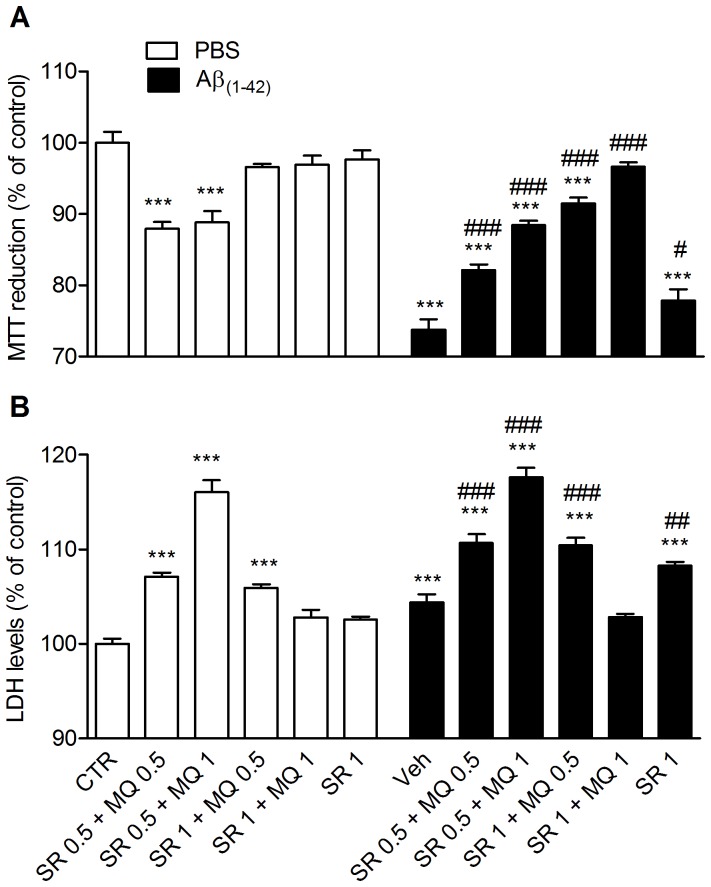
Neuroprotective effect of MQ against Aβ_1–42_ toxicity in rat neuronal cultures. Primary neuronal cultures of rat were simultaneously exposed to 1 µM Aβ_1–42_ and MQ 0.5 or 1 µM after a 24 h sulforaphane (0.5 or 1 µM) pre-treatment. Whereas both the MQ concentrations partially protected from Aβ_1–42_ toxicity following 0.5 µM sulforaphane exposure, the administration of 1 µM MQ after a pre-treatment with 1 µM sulforaphane was fully effective in protecting neuronal cultures from Aβ_1–42_-induced neuronal death. Cell viability was assessed using the MTT (A) or LDH (B) assays. Data are expressed as mean ± S.E.M. Statistical analysis was performed using One-way ANOVA followed by Dunnett's multiple comparison test. *** p<0.001 compared with CTR; ^#^ p<0.05, ^##^ p<0.01, ^###^ p<0.001 compared with Aβ_1–42_.

Finally, we investigated *in vitro* the role of MQ antioxidant activity in counteracting neuronal death induced by Aβ_1–42_. Experiments were carried out in primary cultures of rat cortical neurons, using MTT (3-[4,5-dimethylthiazol-2-yl]-2,5-diphenyltetrazolium bromide) and LDH (lactate dehydrogenase) as markers for mitochondrial toxicity and membrane damage, respectively. We also pretreated cells with sulforaphane, a potent inducer of the NAD(P)H:quinone oxidoreductase (NQO1) enzyme, as reported in previous experiments carried out with SH-SY5Y cells [14], [28] . MQ was cytotoxic on rat cortical neurons, and this cytotoxicity was fully prevented by a 24 h pretreatment 1 µM sulforaphane (see Supporting Information, Figure S4). NQO1 can reduce the quinone moiety of MQ to hydroquinone, which is a potent antioxidant and neuroprotective scaffold. We therefore evaluated the neuroprotective potential of MQ against Aβ_1–42_ in neurons pretreated with sulforaphane. Neurotoxicity was induced by treating cells with Aβ_1–42_ (0.5 µM). Then, cells were treated with sulforaphane (0.5 µM). As shown in Figure 4, Aβ_1–42_ induced significant neurotoxic effects on neurons, in a good agreement with previously reported studies [29],[30]. Exposure of cells to MQ (0.5 µM and 1 µM) partially prevented Aβ-induced neurotoxicity, with an increase of MTT and a reduction of LDH levels. When cells were pretreated with a higher concentration of sulforaphane (1 µM), the administration of 1 µM of MQ fully prevented the neuronal death induced by Aβ_1–42_ (see Fig. 4).

## Discussion

In the present study, combining the scopolamine- and Aβ-induced amnesia models with various behavioral tasks, we investigated the efficacy of MQ in treating cognitive dysfunction. These models were selected as they are based on well-known hallmarks of AD: the cholinergic deficit and Aβ aggregation and toxicity.

Early symptoms of AD include disturbances in short-term episodic memory and problems with attention and spatial orientation [31]. Episodic memory is a type of declarative memory that depends on the ability to remember in a determined temporal and spatial context [32]. It is particularly vulnerable to normal aging and dementia [33]. Spatial memory is a subtype of episodic memory, which stores past event information within the spatio-temporal frame [34]. To evaluate the effect of MQ on short- and long-term spatial memory, we used the T-maze and the MWM [35] models. In both models, scopolamine induced a remarkable decline in cognitive performance, and MQ (15 mg/kg) was able to significantly reverse this effect. To further investigate the effect of MQ on additional types of memory, we tested the compound in the NOR and the passive avoidance models. The NOR protocol is based on the rodents' innate interest in novel objects. This behavior can be modeled in mice either with simple recognition memory tasks or with more complex spatial and episodic-like memory events [36]. Passive avoidance is an amygdala-dependent model and evaluates emotional memory. Passive avoidance has been related to ‘long-term’ or reference memory and has been used to study learning and memory following a stressful stimulus [37]. In these models too, the cognitive impairment was induced by scopolamine, and MQ (15 mg/kg *per os*) pretreatment was able to prevent the cognitive decline.

In both sets of experiments, cognitive impairment was caused by reducing cholinergic transmission *via* blockade of muscarinic receptors. Thus, although very encouraging, the potent AChE effect of MQ makes it difficult to distinguish between the pharmacological effect of the drug (i.e. the anti-AChE activity) and its *pure* nootropic activity.

To expand this investigation, MQ was also tested in the Aβ-induced amnesia model. Aβ causes memory disturbances because of its neurotoxicity. It is unclear how Aβ accumulates in the brain. However, when this accumulation occurs, (proto-)fibrils and plaques are formed which are toxic to neurons. Experimentally, Aβ neurotoxicity can be mimicked *in vivo* by direct i.c.v. injection of the peptide to mice. A clear reduction of cognitive performance occurs over the next few days, as a possible consequence of progressive neurodegeneration [38]. This toxicity model is widely utilized and well-characterized [39]: i.c.v. Aβ infusions can significantly alter the oxidative balance in the brain, causing induction of iNOS and increased levels of peroxidized lipids in the tissue 7 days after treatment [40], [41]. Furthermore, Aβ_1–42_ is the main component of diffuse plaques even in the earliest stages of deposition, and it possesses the most fibrillogenic and aggregating activities among Aβ peptides [42]. These characteristics make this model particularly suitable for the investigation of MQ's anti-oxidant and anti-aggregating properties.

MQ (15 mg/kg) was administered according to a sub-chronic time-dependent treatment schedule. The compound was given once a day for 2, 4, and 6 days [43] and cognitive performance was assessed in the T-maze model (short-term memory) and the passive avoidance task (emotional memory). In both models, the 6-day repeated treatment with MQ exhibited the most beneficial effect against Aβ-induced cognitive impairment. Shorter treatments (2 and 4 days) were effective in the T-maze task only.

These findings indicate that MQ is efficacious in non-cholinergic models of cognitive disturbances, suggesting that the nootropic activity of this compound goes beyond its expected ability to reinstate impaired cholinergic transmission.

The cellular mechanism through which MQ reverses Aβ-induced neurotoxicity and amnesia is not clear at present. Aβ is a major plaque component, and lipid oxidation products can modify Aβ structure, increasing its membrane affinity and accelerating the conversion into toxic oligomers and (proto-)fibrils [44]. MQ has already been shown to decrease plaque number and morphology *in vivo* and exhibit anti-aggregation properties *in vitro* [16]. This anti-aggregation effect of MQ might contribute to the anti-amnesic properties of the compound. MQ has also been shown to serve as a substrate for NQO1. NQO1 reduces the quinone moiety of MQ to hydroquinone, which is a potent anti-oxidant [14], similarly to what is done with NQO1 natural substrate, i.e. co-enzyme Q10. In this respect, MQ has been shown to neutralize free radical activity in SH-SY5Y neuroblastoma cells, but only when cells were pretreated with the NQO1 inducer, sulforaphane [28]. Therefore, a possible additional mechanism contributing to MQ's anti-Aβ effect could be related to its indirect antioxidant properties.

To assess this, we studied the effect of MQ in sulforaphane-pretreated primary cortical neurons exposed to toxic doses of Aβ. In these conditions, MQ dose-dependently and fully reversed Aβ toxicity. Nevertheless, MQ in the absence of NQO1 activity showed intrinsic toxicity on neurons related to two possible mechanisms: i) as a quinone and electrophile, it can react and therefore damage proteins and nucleic acids [45]; ii) as a semiquinone, it can react with O_2_ generating the HOO• radical, which is highly reactive and toxic [46]. This suggests that the NQO1-mediated conversion of MQ to the antioxidant hydroquinone is required to unveil the anti-Aβ effect of MQ in neurons. Interestingly, NQO1 is induced in neurofibrillary tangles and in the cytoplasm of hippocampal neurons of AD patients [47]. More recent studies have revealed that NQO1 regional immunohistochemical staining is mostly localized in astrocytes and neurites surrounding senile plaques in substantia nigra, hippocampus and cortex of AD patients [48]. Wang and colleagues [49] have hypothesized that the NQO1 upregulation may take part to a neuroprotective system activated in response to the AD process.

MQ was developed according to the polypharmacology paradigm [12], [50]. In the present study, MQ was found to be broadly effective *in vivo* on both cholinergic and non-cholinergic types of memory disturbances. Moreover, MQ can be effective against oxidative stress thus offering a therapeutic benefit over existing treatments. Together, the present findings and previous studies indicate that MQ might interfere with AD progression at different levels of the neurodegenerative cascade, thus making it a promising multi-target candidate for addressing the complex nature of AD.

## Supporting Information

Figure S1 **2D structure of the multi-target compound, Memoquin.**
(TIF)

Figure S2 **MQ does not alter motor activity.** Effect of MQ treatment on distance traveled in an open field (A) and on motor coordination in the accelerating rotarod task (B) in mice. MQ (7–15 mg/kg, p.o.) was administered 40 min before testing. Data are expressed as mean ± S.E.M. No significant differences were found (One-way ANOVA).
(TIF)

Figure S3 **β-amyloid_(1–42)_ i.c.v. treatment induces cognitive impairment.** Disruptive effect of an i.c.v. injection of Aβ_(1–42)_ (200–800 pmol/mouse) in the spontaneous alternation (A) and passive avoidance task (B). β-amyloid_(1–42)_ and β-amyloid_(42-1)_ or their vehicle (PBS) were i.c.v. injected 7 and 8 days before T-maze and passive avoidance, respectively. Data are expressed as mean ± S.E.M. Statistical analysis was performed using One-way ANOVA followed by post-hoc Tukey's test. ** p<0.01, *** p<0.001 vs. controls (PBS and Aβ_(42-1)_).
(TIF)

Figure S4 **Sulforaphane pre-treatment prevents MQ neurotoxicity.** Rat primary neuronal cultures were exposed to MQ 0.5 (A, B) or 1 (C, D) µM following a 24 h pre-treatment with different concentrations of sulforaphane (0.1 to 2.5 µM). 1 µM sulforaphane pre-treatment fully inhibited the neurotoxicity of both 0.5 and 1 µM MQ. 2.5 µM sulforaphane treatment caused toxicity even in the absence of MQ. Cell viability was assessed using the MTT (A, C) or LDH (B, D) assays. Data are expressed as mean ± S.E.M. Statistical analysis was performed using One-way ANOVA followed by Dunnett's multiple comparison test. *** p<0.001 compared with CTR; ^###^ p<0.001 compared to MQ 0.5 or MQ 1.
(TIF)
